# Protocadherin-10 is involved in angiogenesis and methylation correlated with multiple myeloma

**DOI:** 10.3892/ijmm.2012.880

**Published:** 2012-01-09

**Authors:** YING LI, ZE-SONG YANG, JUN-JUN SONG, QIONG LIU, JIAN-BIN CHEN

**Affiliations:** 1 Department of Hematology, the First Affiliated Hospital of Chongqing Medical University, Chongqing, P.R. China; 2 Department of Emergency, the First Affiliated Hospital of Chongqing Medical University, Chongqing, P.R. China

**Keywords:** myeloma, angiogenesis, methylation, protocadherin-10

## Abstract

Protocadherin-10 (PCDH10) which is located at 4q28.3, is a member of the cadherin superfamily of cell adhesion molecules. PCDH10 is broadly expressed in normal adult, but nearly undetectable in multiple myeloma (MM) tissues and cell lines. Its promoter methylation was detected in virtually all the silenced or downregulated cell lines. The silencing of PCDH10 could be reversed by pharmacological demethylation, indicating a methylation-mediated mechanism. In the current study, we investigated 44 patients (23 females, 21 males), 77.27% (34/44) of whom presented high methylation of PCDH10. We found no associations between promoter hypermethylation and gender or age at the time of initial diagnosis. We also examined the role of PCDH10 as a mediator of MM cell proliferation, cell cycle progression, and its involvement in angiogenesis. Our results demonstrate that the PCDH10 gene is a target for epigenetic silencing in MM and provide a link between the dysregulation of angiogenesis and DNA methylation.

## Introduction

Protocadherin-10 (PCDH10) belongs to the δ2 subgroup of the protocadherin subfamily. The human PCDH10 gene, also known as OL-PCDH or KIAA1400, is located at 4q28.3 on the long arm of chromosome 4 (1). Cell adhesion proteins are involved in a wide range of roles, including cell sorting and recognition, boundary formation, induction and cell-cell adhesion (1,2). The genes encoding adhesion proteins are perturbed in a multitude of human cancers and have been shown to promote survival, migration and metastasis (3,4).

Current literature shows that genetic and epigenetic deregulation of PCDH10 occurs in a multitude of human cancers. Failure to express PCDH10 may result in loss of inhibition of cell migration, thereby contributing to cancer progression (3–8). In addition, increased angiogenesis has been demonstrated in the bone marrow (BM) microenvironment in hematological malignancies, including multiple myeloma (MM), suggesting a potential pathophysiologic role for angiogenesis in MM (9). MM is a plasma cell malignancy characterized by a tight relationship between tumor cells and the BM microenvironment that supports myeloma cell growth and survival (10). In MM, as in solid tumors, disease progression is characterized by a pre-angiogenic stage of slow tumor progression followed by an angiogenic switch and a subsequent angiogenic stage associated with progressive tumor growth (11). The chick embryo chorioallantoic membrane (CAM) is commonly used for the study of *in vivo* angiogenesis (12,13). Through the effect of PCDH10 in CAM angiogenic assays, we found the PCDH10 acts as a novel tumor suppressor gene involved in angiogenesis.

Although the PCDH10 cDNA was cloned in 2000 and much is known about how PCDH10 functions, the mechanistic basis governing the basal expression of PCDH10 has not yet been fully elucidated (14–16). Here we further identified and characterized the promoter and elements that regulate PCDH10 expression in human MM patient samples and cancer cells.

## Materials and methods

### Patients characteristics

We studied 44 patients (23 females, 21 males), with a median age of 66 years (range 43–78 years)admitted to our Institution between January, 2010 and February, 2011. The diagnosis was established according to standard morphological and immunophenotypic criteria and revised according to the International Staging System (ISS) classification. All patients gave informed consent. The study was conducted according to good clinical and laboratory practice rules and the principles of the Declaration of Helsinki.

### Cell culture and sample collection

The KM3 and RPMI-8226 cell lines, gifts from Jian Hou (The Second Military Medical University, Shanghai, China) were routinely maintained in RPMI-1640 media (Gibco, USA), supplemented with 10% heat-inactivated fetal bovine serum (FBS, Gibco). We obtained all specimens in accordance with the Research Ethics Board of the Hematology Laboratory of the First Affiliated Hospital of Chongqing Medical University (Chongqing, China).

### 5-Aza-2′-deoxycytidine and trichostatin A treatment

For the treatment combining 5-aza-2′-deoxycytidine (Aza; Sigma, USA) and trichostatin A (TSA; Sigma), cells were treated with Aza (10 μM) for 3 days and subsequently with TSA (100 ng/ml) for 24 h as previously described (17).

### Total-RNA isolation and semi-quantitative reverse transcription (RT-PCR)

Following tumour sample homogenization, RNA was extracted using the TRIzol reagent (Invitrogen). cDNA was then synthesized using Go-Taq (Promega, USA) and random hexamer primers. β-actin served as a control for RNA integrity. PCDH10 expression was analyzed by PCR. The primers used were PCDH10-F, 5′-ACT GCT ATC AGG TAT GCC TG-3′ and PCDH10-R, 5′-GTC TGT CAA CTA GAT AGC TG-3′; β-actin-F, 5′-CTC CAT CCT GGC CTC GCT GT-3′ and β-actin-R, 5′-GCT GTC ACC TTC ACC GTT CC-3′. RT-PCR was performed for 32 cycles for PCDH10 and 23 cycles for β-actin.

### DNA bisulfite treatment and methylation analysis

DNA was extracted from MM and healthy adult BM samples by a standard protocol (Zymo Research, USA). Bisulfite modification of DNA and the methylation status in the CpG islands of the PCDH10 promoter were carried out as previously described (3). PCDH10 primers detecting methylated (M) or unmethylated (U) alleles of the PCDH10 promoter were: PCDH10-M1, 5′-TCG TTA AAT AGA TAC GTT ACG C-3′ and PCDH10-M2, 5′-TAA AAA CTA AAA ACT TTC CGC G-3′ for methylated alleles; PCDH10-U1, 5′-GTT GTT AAA TAG ATA TGT TAT GT-3′ and PCDH10-U2, 5′-CTA AAA ACT AAA AAC TTT CCA CA-3′ for unmethylated alleles. Methylation-specific PCR (MSP) was performed for 40 cycles using Ampli Taq-Gold (methylation-specific primer, annealing temperature 600˚C; unmethylation specific primer, annealing temperature 580˚C). MSP primers were first checked for not amplifyling any unbisulfited DNA and the specificity of MSP was further confirmed by direct sequencing of some PCR products. PCR reactions were resolved on a 2% agarose gel.

### Construction of expression plasmids

pcDNA3.1(+)TP53 was constructed by subcloning the full-length wild-type TP53 from plasmid pC53-SN (a gift of Bert Vogelstein) into pcDNA3.1(+). pcDNA3.1(+)PCDH10 was constructed by cloning the PCR product generated from the full-length clone of KIAA1400 (gift from Kazusa DNA Research Institute, Japan) with AccuPrime Pfx DNA Polymerase (Invitrogen). All the plasmid sequences and orientations were confirmed by sequencing.

### Colony formation assays

For the colony formation assay using monolayer culture, cells (2×10^5^/well) were plated on a 6-well plate and transfected with expression plasmids or the empty vector (2 μg each), using Lipofectamine 2000 (Invitrogen). Cells were collected and plated in a 5-cm dish 48 h post-transfection, and selected after 21 days with G418 (0.4 mg/ml). Surviving colonies (50 cells/colony) were counted under a fluorescence microscope.

For the colony formation assay using semi-solid medium, cells were transfected as above. At 48 h post-transfection, cells were suspended in RPMI-1640 containing 1% methyl cellulose, 35% FBS and 0.8 mg/ml G418 in a 5-cm dish. The dish was placed in a sealed chamber and incubated at 37˚C in a 5% CO_2_ incubator for 21 days. The number of colonies/cm^3^ was counted under an inverted microscope. Total-RNA from the transfected cells was extracted, treated with DNAse I and analyzed by RT-PCR and western blotting to confirm the ectopic expression of PCDH10. All the experiments were performed in triplicate wells three times.

### Cell cycle analysis

PCDH10-8226 or Vector-8226 cells were cultured in RPMI-1640 medium and 10% FBS with G418 (0.4 mg/ml). These cells were harvested and fixed in ice-cold 70% ethanol for 1 h. The cell cycle profiles were assayed by the Elite ESP flow cytometer and data were analyzed with the CellQuest software (BD Biosciences, USA).

### Protein extraction and western blot analysis

For western blot analysis, total cellular extracts were obtained by lysis of cells in a lysis buffer and a protease inhibitor cocktail. Protein concentrations of the cell lysates were determined by the Bradford method (Bio-Rad, Hercules, CA). An equal volume of 2X sodium dodecyl sulfate (SDS) loading buffer was added, and the samples were boiled for 5 min. Protein samples (70 μg/lane) were separated by SDS-polyacrylamide gel electrophoresis (SDS-PAGE) and transferred to nitrocellulose filters (Amersham Biosciences, Piscataway, NJ). The filters were blocked with Tris-HCl buffer saline containing Tween-20 buffer (pH 7.6, 10 mM Tris-HCl buffer, 0.15 M NaCl and 0.05% Tween-20) and 5% skim milk at room temperature for 1 h and then incubated with the primary antibody at 4˚C overnight [1:800 dilution of mouse monoclonal antibody against human PCDH10, obtained from Abnova; 1:1,000 mouse monoclonal antibody against glyceraldehyde-3-phosphate dehydrogenase (GAPDH), Epitomics, Inc., USA], followed by the addition of a horseradish peroxidase-conjugated antibody (Cell Signaling Technology, 1:2,000). The bands were visualized using the enhanced chemiluminescence substrate (Cell Signaling Technology).

### Chick chorioallantoic membrane (CAM) assays

White fertilized eggs (56–64 g) with a surface free of pathogens and without damage were incubated for 8 days. The eggs were placed in a 37˚C incubator upward and allowed to hatch for 24 h, ensuring 40–60% relative humidity. The eggs were rotated each morning and evening.

On Day 9, the chick embryos were randomly divided into 4 groups of 3 embryos each. The chick embryo chorioallantoic membrane surface spaces (avoiding the vascular chorioallantoic membrane) were respectively exposed to 100 μl RPMI-1640 medium (control group), 100 μl of transfected RPMI-8226 cell culture supernatant (positive control group), 100 μl of transfected PCDH10-treated RPMI-8226 cell culture supernatant (transfection group) or 100 μl of RPMI-8226 cell culture supernatant after transfection with empty plasmid (empty plasmid group), at 38˚C for 48 h. The sample fluid was extracted from 1×10^6^ cells cultured for 72 h, followed by freezing and dehydration, and cells were dissolved in 2 ml RPMI-1640 and filtered. All operations were strictly aseptic.

After 12 days exposure of the embryos to test material, the chorioallantoic membrane was removed and fixed to allow the observation of the vascular zone. The vascular networks in the CAMs were blindly scored for the presence or absence of an avascular zone larger than 5 mm.

### Statistical analysis

All statistical calculations were performed using SAS version 3.1 for Windows (SAS, Institute, USA). The results are expressed as values of mean ± SEM. Differences between the subgroups were tested with the 2-tailed t-test, P-values <0.05 were considered to indicate significant differences.

## Results

### PCDH10 downregulation and promoter hypermethylation in MM cell line

**Figure 1:**
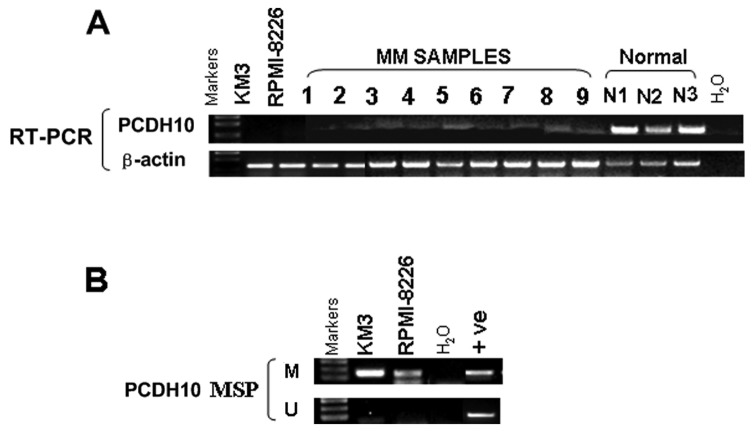
(A) Downregulation of PCDH10 in the MM cell lines KM3, RPMI-8226 and in MM samples and PCDH10 overexpression in human normal samples determined by semi-quantitative RT-PCR, with β-actin as a control. (B) PCDH10 expression and methylation status in KM3 and RPMI-8226 cells was determined by methylation-specific PCR (MSP) analysis. M, methylated; U, unmethylated.

To examine if PCDH10 is downregulated in MM, we first examined its expression in KM3 cells, 3 normal adult BM samples and 9 MM samples by semi-quantitative RT-PCR. PCDH10 was silenced in MM cells and 9/9 (100%) MM samples while it was readily detected in normal adult BM samples (0/3) (Fig. 1A). The PCDH10 CpG island (CGI) was methylated in KM3 and RPMI-8226 cells (Fig. 1B), while no methylation was detected in normal adult BM (Fig. 3B), samples showing that silencing of PCDH10 expression in MM was correlated with its methylation status.

### Pharmacological demethylation reactivates the silenced PCDH10

**Figure 2:**
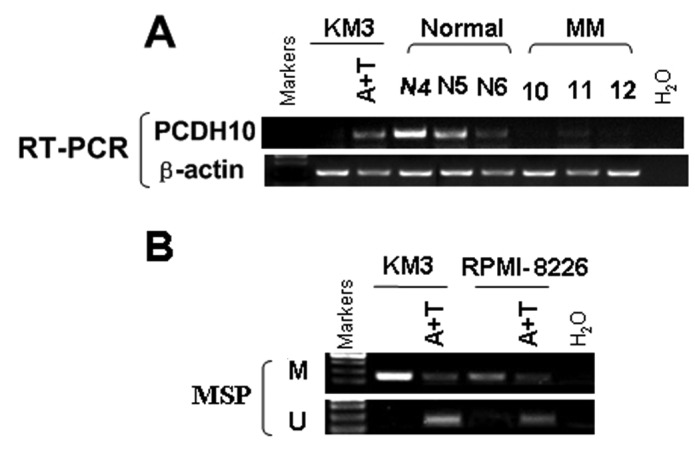
Detection of unmethylated PCDH10 alleles (U) in the MM cell lines KM3 and RPMI-8226, by methylation-specific PCR (MSP) analysis after 5-aza-2′-deoxycytidine (A) and trichostatin A (T) treatment. M, methylated; U, unmethylated.

To evaluate the effect of promoter CGI methylation on the expression of PCDH10, KM3 and RPMI-8226 cells were treated with a DNA methytransferase inhibitor, Aza for 3 days together with a histone deacetylase inhibitor TSA for 1 day. PCDH10 mRNA expression was dramatically induced after the treatment. This reactivation was associated with an increase of unmethylated alleles and a decrease of methylated alleles of the PCDH10 promoter, as assessed by MSP (Fig. 2). These results show a direct link between CGI methylation and PCDH10 silencing.

### Hypermethylation and disease evolution

**Figure 3:**
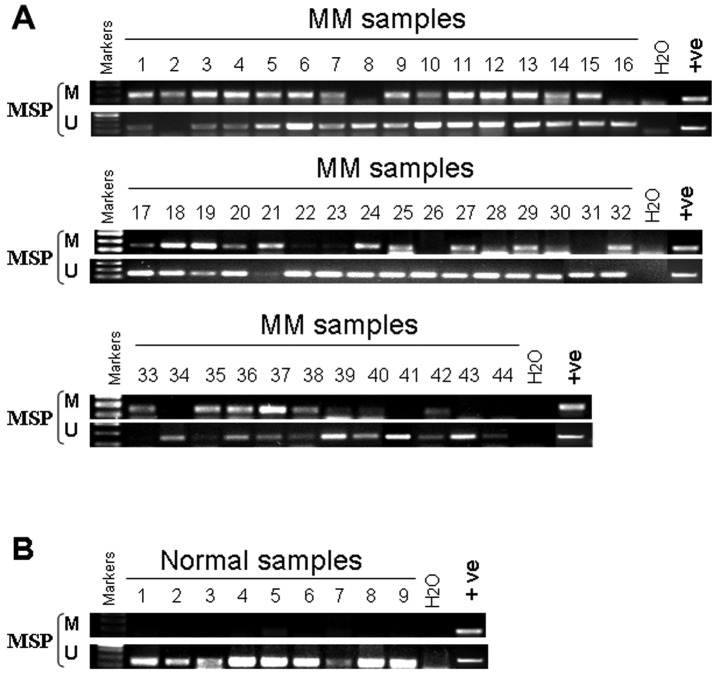
PCDH10 CpG methylation in MM and normal adult bone marrow samples. (A) MM bone marrow samples. (B) Normal adult bone marrow samples. M, methylated; U, unmethylated.

We examined the dynamics of PCDH10 hypermethylation during disease evolution in MM. We examined sequential samples from 44 patients at diagnosis and after a median of 12 months (range 1–30 months), (Fig. 3A) either during routine follow-up (n=6), or in the presence of disease progression (n=8), with blast counts increasing from 6.9±1.7 to 31.7±7.5% (mean ± SEM, P<0.05). We found that the methylation status of PCDH10 did not change (Table I).

### Ectopic expression of PCDH10 inhibits tumor cell clonogenicity and induces G1 cell cycle arrest

**Figure 4:**
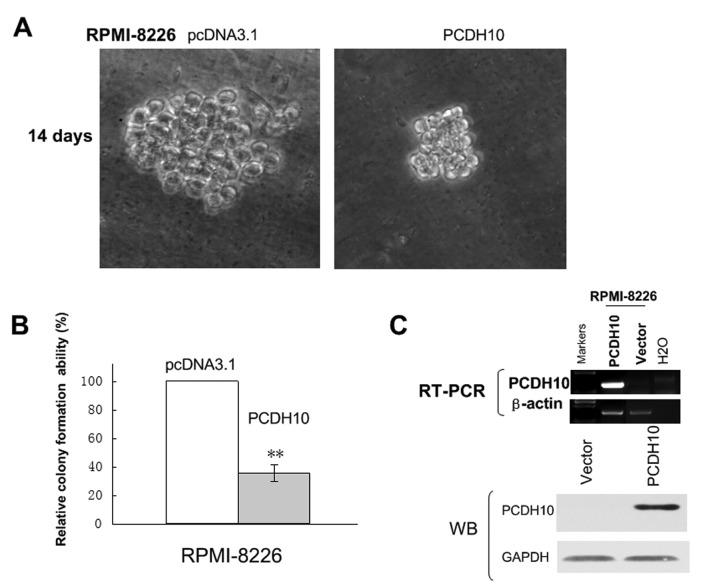
Ectopic expression of PCDH10 inhibited tumor cell clonogenicity. (A) Representative inhibition of colony formation in soft agar culture by PCDH10. (B) Quantitative analysis of colony formation. The numbers of G418 selection colonies in vector-transfected cells were set to 100%, values are mean ± SD, P-values were calculated using the Student's t-test. The asterisk indicates statistical significant difference (P<0.05). Independent experiments were performed in triplicate wells for three times. (C) Ectopic expression of PCDH10 at the transcript level in transfected RPMI-8226 cells was confirmed by RT-PCR and western blotting.

To evaluate the role of PCDH10 as a tumor suppressor gene (TSG) in MM, we thus sought to establish whether ectopic expression of PCDH10 could inhibit tumor cell clonogenicity. The expression vector encoding full-length PCDH10 or vector alone were transfected into RPMI-8226 cells, in which PCDH10 was fully silenced by methylation. After G418 seletion for 3 weeks, stable overexpression of PCDH10 as shown by RT-PCR and western blotting, was successfully obtained (Fig. 4C).

A colony formation assay (Fig. 4A) was used to evaluate the suppressor function of PCDH10 in vector of PCDH10-transfected cells. Ectopic expression of PCDH10 dramatically reduced the colony formation efficiencies of RPMI-8226 cells (Fig. 4B) down to 40–50% of vector controls (P<0.05), indicating that PCDH10 functions as a TSG in KM3 and RPMI-8226 cells.

**Figure 5:**
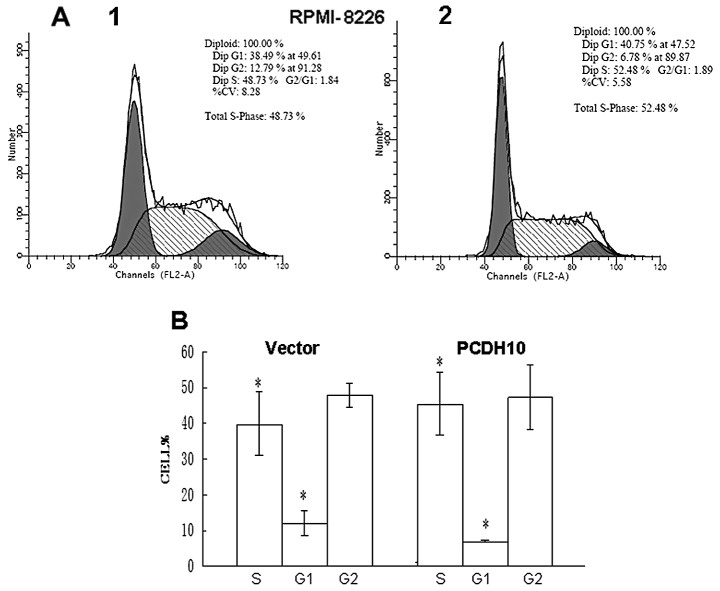
PCDH10 induces cell cycle arrest at the S phase in RPMI-8226 cells. (A) Representative distribution of cell cycles in RPMI-8226 cells transfected with or without PCDH10 by fluorescence-activated cell sorting analysis. (B) Analysis of the distribution of RPMI-8226 cells transfected with or without PCDH10 in different phases of cell. Results are presented as average ± SD (n=4) statistical significance was evaluated by the Student's t-test (P<0.05).

To further explore the mechanism by which PCDH10 suppresses colony formation, we investigated the effect of PCDH10 on cell cycle distribution by flow cytometry. The percentage of cells in the G1 phase was increased in PCDH10-transfected cells compared with vector RPMI-8226 cells (P<0.05), indicating that the effect of PCDH10 was likely to be independent of the cell cycle (Fig. 5). Thus, collectively the results demonstrate that PCDH10 has growth inhibitory activity and is a functional TSG in MM.

### Overexpression of PCDH10 has tumor inhibitory effect on the colony formation of tumor cells

The frequent silencing of PCDH10 in MM cell lines, compared with its broad expression in normal tissues, suggests that PCDH10 might have tumor suppressor function. We investigated whether restoration of PCDH10 could suppress the clonogenicity of MM cell lines RPMI-8226 which are methylated and silenced for PCDH10. Two weeks after transfection and subsequent selection of drug resistant colonies (18,19), the numbers of colonies produced by PCDH10-transfected cells was significantly less than that by empty vector-transfected cells, suggesting that PCDH10 does suppress the colony formation of tumor cells (Fig. 5).

### Chick chorioallantoic membrane (CAM) assays

Addition of a PCDH10 gene transfection cell culture supernatant in chick embryo CAM and comparison of the vascular map between this transfection group and the negative control group, indicated that the vascular branches decreased. On the other hand, in the positive control group and the blank control group, vascular branch growth increased, and abnormal shapes were observed resulting in abnormal vascular network structure.

**Figure 6:**
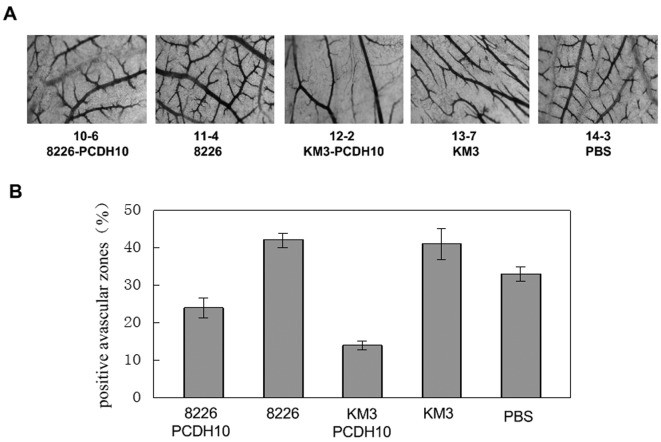
Inhibition of neovascularization in the chorioallantoic membrane (CAM) by PCDH10. Thermanox coverslips containing PBS or a range of concentrations of Vector or PCDH10 were applied to the CAMs of 3-day-old chick embryos. After 48 h, the formation of avascular zones (>5 mm in diameter) was analyzed. (A) Photographs of representative CAMs given Thermanox plates containing PBS, Vector or PCDH10. (B) Dose-dependent inhibition of angiogenesis in the CAM by PCDH10. Shown are the percentages of the number of CAMs showing an avascular zone relative to the total number of CAMs assessed.

Statistical analysis show that the chick embryo chorioallantoic membrane vascular branching points, in the blank group and positive control group compared with the control and transfection groups increased about 40–60% (Fig. 6).

The total length of the vascular branches in the transfection group compared with the control group decreased by 43% (RPMI-8226) and 66% (KM3), lower than that in the blank group and positive control group. The increases in the positive control group compared with the blank group were approximately 21 and 20% (Fig. 6). The branch vessel total length of the positive control group and blank group significantly increased compared to that of the transfection group. The results show that, PCDH10 can inhibit the growth of chick embryo chorioallantoic membrane angiogenesis.

## Discussion

In this study, we found that PCDH10, located at an important tumor suppressor locus 4q28.3, was totally silenced in KM3 and RPMI-8226 cell lines, but widely expressed in normal adult bone samples. PCDH10 silencing in cancer cell lines was well correlated with its promoter CpG methylation, which could be restored by pharmacological demethylation, suggesting that promoter methylation of PCDH10 plays an important role in its inactivation in MM (6–8). Moreover, in patients with MM, there were no associations between hypermethylation and clinical characteristics, including IPSS score, ISS classification and cytogenetics. Whether this suggests that in early onset MM, the methylation of PCDH10 may have changed, or it indicates a change predicting the pathogenesis of MM, remains to be elucidated. The present study has proven that PCDH10 is an active member of the MM gene methylation profiling.

We located the core promoter of PCDH10 to a 462-bp segment of the 5′-flanking region characterized by a high GC content. It has also been reported that Sp1/Sp3 and CBF/NF-Y transcription factors play a crucial role in the basal expression of the human PCDH10 gene (4). Gene-specific hypermethylation was observed at the myeloma stage (20). Methylation was evident at the development of myeloma, particularly of genes involved in cell-cell signaling and cell adhesion, which may contribute to independence from the BM microenvironment. More specifically, methylation subgroups defined by translocations and hyperdiploidy were found, with t(4;14) myeloma having the greatest impact on DNA methylation (21,22). Thus as one of the significant cadherins, PCDH10 may have the potential to be a epigenetic regulator.

Substantial evidence has shown that tumor growth and metastasis are angiogenesis-dependent (12,13,23). In MM, the increased BM angiogenesis is due to the aberrant expression of angiogenic factors by myeloma cells, the subsequent increase in pro-angiogenic activity of normal plasma cells as a result of myeloma cell angiogenic activity, and the increased number of plasma cells overall. Molecular mechanisms underlying the angiogenic switch in MM have recently been identified. The BM microenvironment is hypoxic and the hypoxia induced transcription factor HIF-1α is critically involved in the production of angiogenic factors by myeloma cells. In addition, myeloma cells, stem and progenitor cells directly produce or induce several pro-angiogenic molecules in the microenvironment, including VEGF, bFGF, Ang-1, OPN, HGF, HOXB7, IL-8, and PGE2 (9,24,25).

This complex pathogenesis of myeloma-induced angiogenesis suggests that several pro-angiogenic molecules and related genes in the myeloma microenvironment are potential therapeutic targets (9). Our results show that PCDH10, an important member of the cadherin family, may directly induce several pro-angiogenic molecules in the microenvironment, and plays a positive role in inhibiting tumor angiogenesis. This indicates that silencing of a tumor suppressor may be implicated in its role in angiogenesis, and the recovery of some tumor suppressor gene expression may inhibit angiogenesis so as to exert the tumor suppressor function (26,27). It is also possible that epigenetics and angiogenesis have a potential connection, worthy of further research and discovery.

Vascular disrupting agents (VDAs) target the cytoskeleton and tubulin network of endothelial cells, thereby causing vascular disruption and subsequent tumor cell death (12,13). Moreover, the novel VDA, plinabulin, inhibits tumor growth in human plasmacytoma mouse xenograft models, at well-tolerated doses. These studies provide the rationale for the development of plinabulin as a novel therapy to improve patient outcome in MM (23,28).

Treatment modalities targeting genes perturbed by epigenetic mechanisms, such as decitabine, have entered clinical trials for cancers, such as chronic myeloid leukemia, and other myelodysplastic syndromes (29–32). As genome-wide approaches begin to unravel the epigenomic landscape of MM, the utility of targeting genes and pathways deregulated by epigenetics will be uncovered (20,32). In the present study, we have shown that PCDH10 represents a tumour suppressor gene in MM which may benefit from such an approach. Moreover, PCDH10 involved in angiogenesis, with its epigenetic silencing correlated with MM. Thus, further study of the molecular mechanism of PCDH10 is necessary, and will be the focus of subsequent research in our laboratory.

## Figures and Tables

**Table I:** Correlation between PCDH10 gene methylation and the clinical characteristics of MM patients.

	Status of PCDH10 methylation			
			
Patient parameters	Methylated (n=34)	Unmethylated (n=10)	Total (n=44)	P-value
Age at diagnosis				
Mean ± SD	62.3±9.4	68.7±6.9		
Range	54–77	43–78	43–78	0.074
Gender, n (%)				
Female	19 (43.2)	4 (9.1)	23 (52.3)	
Male	15 (34.1)	6 (13.6)	21 (47.7)	
ISS stage, n (%)				0.153
I	12 (27.3)	3 (6.8)	15 (34.1)	
II	15 (34.1)	3 (6.8)	18 (40.9)	
III	7 (15.9)	4 (9.1)	11 (25)	
Platelets (×10^9^)	162±77	231±86		0.885
Hemoglobin (g/l)	95.7±23.0	93.2±25.2		0.800
Serum calcium (mg/dl)	2.23±0.39	2.24±0.20		0.599
Serum creatinine (SCr.) (mg/dl)	128.0±124.9	117.0±116.3		0.801
